# Anti-Runaway Prevention System with Wireless Sensors for Intelligent Track Skates at Railway Stations

**DOI:** 10.3390/s17122955

**Published:** 2017-12-19

**Authors:** Chaozhe Jiang, Yibo Xu, Chao Wen, Dilin Chen

**Affiliations:** 1 National United Engineering Laboratory of Integrated and Intelligent Transportation, Chengdu 610031, China; jiangchaozhe@163.com; 2 Railway Research Center, University of Waterloo, Waterloo, ON N2L 3G1, Canada; 3 Cloud-Guizhou Big Data Science Application Research Center, Guiyang 550081, Guizhou, China; yibo.xu@csdata.com.cn; 4 Department of Industrial and Enterprise System Engineering, University of Illinois at Urbana-Champaign, Urbana, IL 61801, USA; dilin2@illinoise.edu

**Keywords:** anti-runaway prevention, intelligent track skates, detecting and communicating sensor, wireless communication technology, real-time monitoring and management system

## Abstract

Anti-runaway prevention of rolling stocks at a railway station is essential in railway safety management. The traditional track skates for anti-runaway prevention of rolling stocks have some disadvantages since they are operated and monitored completely manually. This paper describes an anti-runaway prevention system (ARPS) based on intelligent track skates equipped with sensors and real-time monitoring and management system. This system, which has been updated from the traditional track skates, comprises four parts: intelligent track skates, a signal reader, a database station, and a monitoring system. This system can monitor the real-time situation of track skates without changing their workflow for anti-runaway prevention, and thus realize the integration of anti-runaway prevention information management. This system was successfully tested and practiced at Sunjia station in Harbin Railway Bureau in 2014, and the results confirmed that the system showed 100% accuracy in reflecting the usage status of the track skates. The system could meet practical demands, as it is highly reliable and supports long-distance communication.

## 1. Introduction

Safety is of profound significance in railways; for instance, the safety of trains running, the safety of rolling stock or wagons deaccelerating and stopping, safety monitoring, and anti-runaway prevention are few of the main operations at railway stations. For these reasons, many apparatuses are used at stations, particularly for stopping and anti-runaway prevention. Hand brakes, which are widely used in American railway marshaling stations, have strict application conditions; for instance, the wagon-type must be in accordance with the handbrake type, and the hand brakes should be handled manually [1,2]. Although, wheel chocks, which are also called brake skates since they work similar towheels of skates, are mainly handled manually, they perform better than hand brakes in wagon braking and stopping [3]. Track skates can increase the friction between the wheels and tracks and can stop the railway wagons, and, thus, can be used for railway wagon anti-runaway prevention [4,5]. In Italian railways, brake skates are among the most popular devices used for wagon braking and stopping, owing to their simple structure, low cost, and effectiveness [6]. In Chinese railways, the most popular anti-runaway prevention apparatus is the so-called track skates, which was brought in from Russia in the 1940s. Because of their ease of use, less power consumption, and economical and practical applications, track skates are widely used at all stations for anti-runaway prevention of rolling stocks. Track skates can be placed and moved conveniently, preventing rolling stocks’ anti-runaway effectively, and therefore, play a very significant role in railway safety [7]. 

When rolling stocks are stopped on the tracks at stations where they are meant to stay for a long while, the handbrakes at the end of all departure tracks should be pulled, and the rolling stocks must be firmly fixed with the manually operated track skates. However, the real-time usage status of track skates is generally recorded manually by the yardmaster, who is responsible for recording all information of the yard at the stations. The information is displayed in the yard control room, which can inform the staff concerned about track skates about the rolling stocks’ operation status according to a series of labels attached to the information board. Even though the track skates’ usage status is carefully double-checked, there can be errors due to negligence, leading to serious accidents. If an unsuccessful anti-runaway prevention occurs, the rolling stocks may crash into other trains, resulting in casualties and major property damage. For instance, in 2007, the freight train 85209 originating from Lanzhou Railway Bureau slipped because of the misplaced track skates, and collided with passenger train N857, which stopped right after it. This accident caused the derailment of nine rolling stocks, luckily without hurting any passenger. In another instance, a freight train, with 47 rolling stocks, stayed in Yunnan Xuanwei Xiaoji Street station on 4 February 2008, and anti-ran into the direction of Guiyang and caused major casualties while crashing into the houses near the railway station. Another accident took place in 2013 in Golmud train station three at the Xining to Golmud section. A parked freight train waiting for marshaling slipped away to the Garham-Golmud station and collided with the passing train 7581, causing 50 injuries and one death.

To reinforce the safety operations of anti-runaway prevention, each station is equipped with a strict safety inspection system and an anti-slipping inspection team, which checks the anti-runaway prevention status and ensures proper operation of the system. However, because of a lack of necessary technology-monitoring measures for safety management, the anti-runaway safety checks can only rely on humans’ subjective consciousness and their sense of responsibility. Recently, loss or improper use/installation of track skates has caused a derailment, leading to frequent wheel slipping. Due to the widely distributed railway networks in China and several rolling stocks staying at railway stations every day, the management of the anti-runaway track skate system is a significant problem for railway operators.

Since a sensor with high-precision measurement of temperature, strain, vibration, and birefringence can find applications in almost all types of devices, it is increasingly popular in the railway industry [8]. The use of sensors in applications such as detection of driver drowsiness [9], detection of gases with low adsorption energy [10], and measurement and analysis of train motion and railway track characteristics has been researched recently [11]. According to a survey on wireless sensors for condition monitoring in the railway industry [12], a broad range of sensors is used in railway monitoring to provide an extensive range of data and allow monitoring of different structures, vehicles, and machinery. In addition, the use of wireless sensornetworks inrailwayhas shown remarkable development in reducing human and material losses [13]. To enable decision support in the railway infrastructure maintenance lifecycle, the sensor data from wireless sensornetworks need to be translated into relevant and clear information. Using a wireless real-time communication system, the train integrity functionality can be strongly enhanced [14]. Reviewing the literature, we found that the operation of manually monitored, traditional track skates faces several severe problems. First, it is difficult to make sure that the track skates are placed properly. Second, the anti-runaway prevention information needs to be recorded, transferred, and handled manually, which has many potential security risks. Third, the track skates are likely to get stolen due to the open and complexity circumstances. Consequently, it takes much more staff to secure the railway cargo anti-runaway, especially in China. This lag in the anti-runaway prevention mechanism mismatches the other advanced railway technologies and is one of the bottlenecks constraining the overall service level of the railway.

To bridge this gap, we developed a new anti-runaway prevention system (ARPS) based on intelligent track skates, which are equipped with detection and communication sensors, using the technologies of wireless communication sensors. This system focuses on monitoring the real-time status of the track skates and is applicable to arrival and departure tracks with slopes as well as specialized lines with a steep slope and other station tracks with a potential slipping due to the natural environment. Without changing the original track skate operation process, this system applies a radio frequency network and the developed micro-power wireless communication module to transmit information that is detected and collected by the sensors installed in the track skates, to realize automatic recognition of the installation/uninstallation status of the track skates. The real-time status of the track skates can be dynamically shown on the screen at the control center, using the real-time monitoring and management system. ARPS prevents accidents effectively, such as derailment and anti-runaway caused by loss or improper use/installation of track skates, and, thus, enhances safety and avoids errors caused by negligence in the operation of railway cargo anti-runaway prevention. 

The remainder of this paper is organized as follows. The improvements in anti-runaway prevention based on the intelligent track skates equipped with detecting and communicating sensors are depicted in Section 2. The design of intelligent track skates is discussed in Section 3. The design of a real-time monitoring and management system is proposed in Section 4. The test and practices are described in Section 5. The conclusions are summarized in Section 6.

## 2. Improvements in Intelligent Track Skates Equipped with Sensors

The main concept of improvements made in intelligent track skates is to boost the current anti-slipping apparatus without changing the physical operation processes of the current track skates, and simplify the operation of the railway staff. The operation process of the current track skates is as follows:

(1) To set the track skates, the shunting warden initially gives instructions to the shunting staff after the rolling stocks stop at the reserved spots, and then the staff take the track skates from the custodian at the station and reach the specialized track to install the track skates after being notified about the track skate installation. During this process, the shunting team informs the shunting warden face-to-face or via radio about the completion of the installation, and the shunting warden draws a hand-painted skate symbol on the station line board.
(2) To release (move) the track skates, the shunting warden informs the shunting team to take down the track skates from the track, and then erases the related symbol on the information board after the trains have departed from the station.

The mentioned operation process above has apparent shortcomings, the most important being the interaction safety affirmation between the staff. The shunting warden might not obtain the status information of the missive installation or release of the track skates, or might not be aware of the lost track skates, which may result in serious accidents.

**Figure 1:**
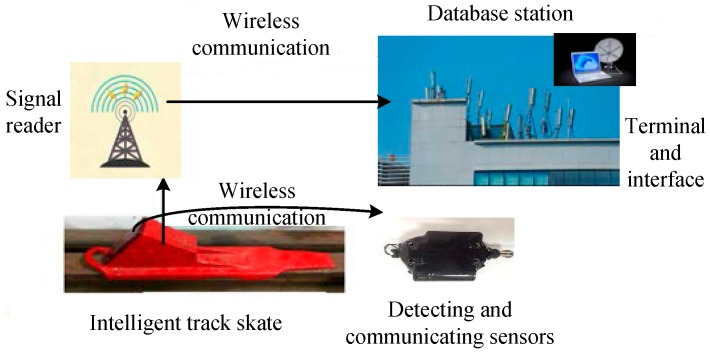
Composition and the work process of anti-runaway prevention system (ARPS).

Using the intelligent track skates equipped with detecting and communicating sensors, a double-check closed-loop ARPS based on the intelligent track skates was established, whose composition and work process are shown in Figure 1. In this system, four modules, including intelligent track skates with detecting and communicating sensors, a signal reader, a database station, and a monitoring system, realize the basic functions from the following three aspects: 

First, for preventing accidents caused by improper use/installation of track skates, the system sends information about track occupation status through the sensors installed in the intelligent track skates after installation completion. The shunting warden can double-check the usage status of the track skates in the monitoring system after the reporting of successful placement by the system. The shunting warden can also input and alter the information about the real-time status of parked rolling stocks provided by other shunting staff, and this double-check action will make it easier for the station signalmen and other related staff to know the real-time status of the entire station.

Second, for accidents caused by improper uninstallation of track skates by the shunting team, the sensors can send off-track signals when uninstallation of track skates occurs. The shunting warden will receive signals according to the abnormal status of the track skates and check the relative on-track status of the track skates. The intelligent track skates equipped with detecting and communicating sensors can provide real-time on-track information when track skates are placed properly. The sensors will trigger the on-track inspection switch with its own gravity, and connect the lithium batteries with a wireless transceiver circuit. Then, the state of the wireless transceiver circuit will change from sleep to work, and the microcontroller chip in the track skate will send an on-track signal to the wireless communication module. After obtaining the direct-sequence spread spectrum and high-frequency modulation by the amplifier circuit, the wireless communication module will send the required/related data to the reader. The sensors of the wireless communication system form the core module of the intelligent track skates, since the performance of the track skates mainly depends on the operation efficiency and accuracy of the sensors. Only when the sensors are installed with the effective wireless communication module canthe data collected in the marshaling yard be transmitted with high efficiency. 

Third, in the case the on-track skates are stolen or do not work in the required state for unknown reasons, the shunting warden can receive signals timely. Then, the shunting warden can arrange relative inspections with the staff according to the abnormal situation of the track skates, and, correspondingly, respond to the inspection results to avoid potential accidents. With the closed-loop status of the track skates, the correct layout and work status can be guaranteed.

## 3. Design of Intelligent Track Skates

### 3.1. Location of the Detecting and Communicating Sensors

**Figure 2:**
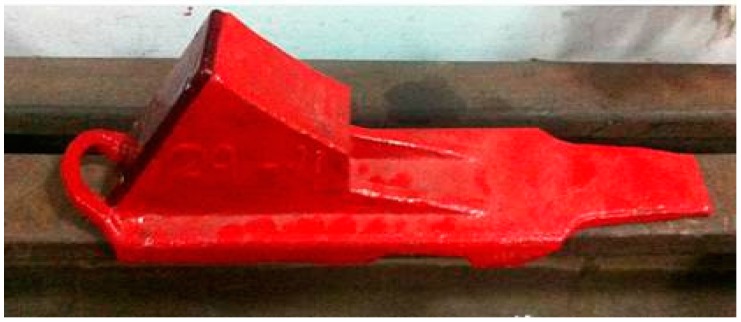
The terminal of intelligent track skate shell.

**Figure 3:**
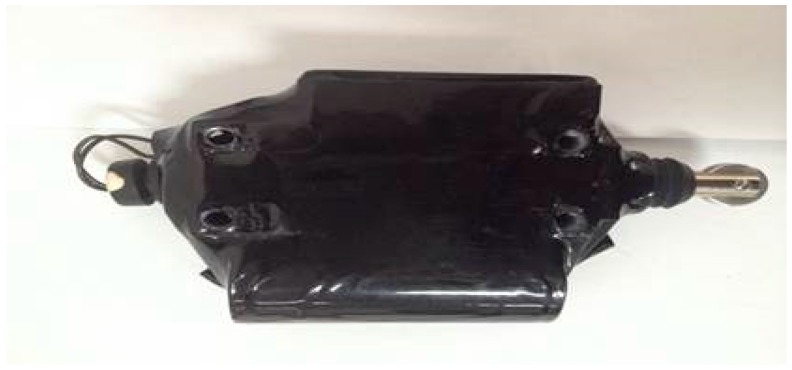
Detecting and communicating sensor of intelligent track skates.

**Figure 4:**
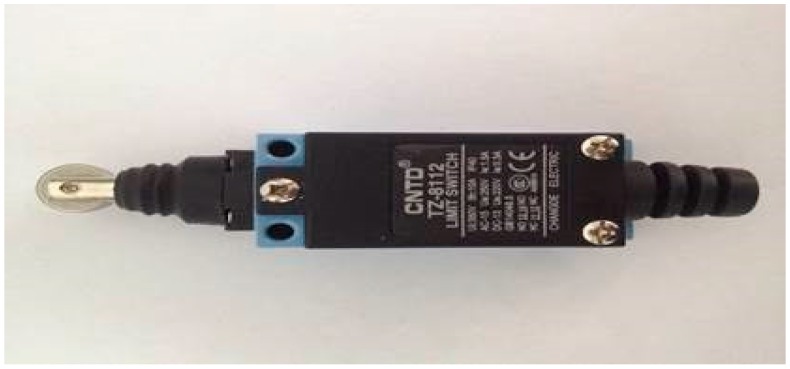
On-track inspection travel switch.

**Figure 5:**
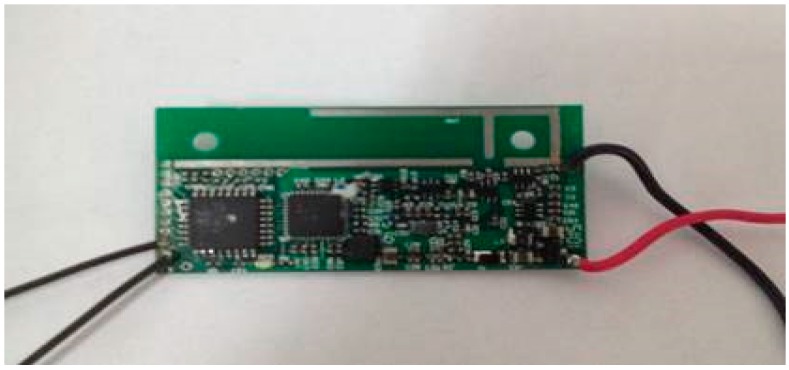
Wireless transceiver circuit.

**Figure 6:**
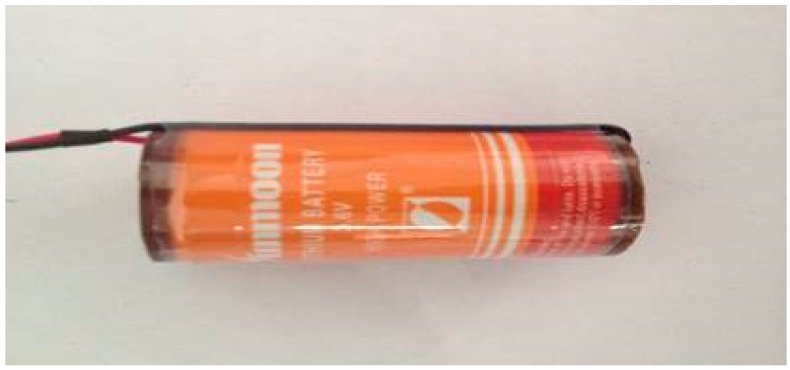
**A** 7000mAh/3.6 V lithium battery.

The intelligent track skates comprise two parts: track skate shells and the status detecting and communicating sensors (Figure 2 and Figure 3, respectively). The other parts shown in Figure 4, Figure 5 and Figure 6 form the enclosure of the sensors, including a travel switch for on-track inspection of a wireless transceiver circuit and a 7000 mAh/3.6 V lithium battery.

The main problem faced by this design is how to distinguish the status of the track skates using the sensors. After repeated experiments and a series of tests, we decided to use an on-track inspection travel switch to serve as the trigger of the detecting and communicating sensors (Figure 4). Through the contacted and separated states between the mechanical parts, the switch realizes the goal of connection or disconnection for the circuit. The roller travel switch can monitor the on-track status of the track skates and roll with the track skates when the wheels start running, without damaging the tracks. When the track skates are placed on the tracks, the travel switch will be closed by the extrusion caused by both tracks and tread of the track skates. When the track skates are taken down from the tracks, the switch will be in the off state. Since the travel switch does not require any power supply, it will not increase the power consumption of the system.

**Figure 7:**
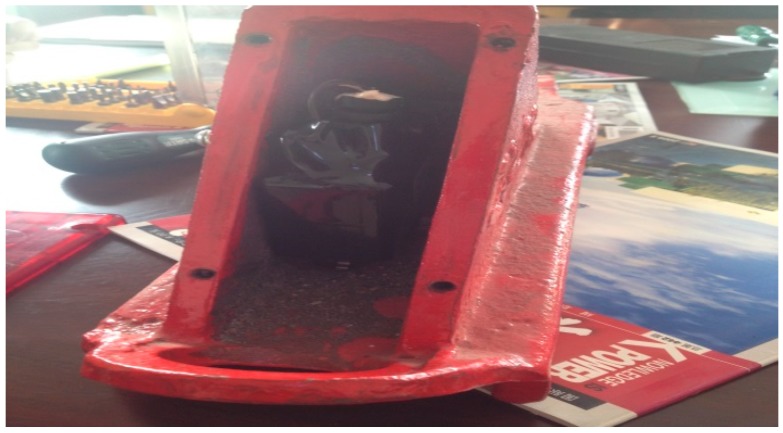
The position of the sensor and its enclosure of a track skate.

To meet the standards of the track skates used in the railway industry in China, the detecting and communicating sensors and the control platform in the monitoring center are embedded in the track skates, as shown in Figure 7. In addition, the detecting and communicating sensors, as well as the enclosure, are finally decided to be placed between the track skate tread and the vertical plates. In this scheme, the sensors will be under the protection of both vertical plates and the stepped surface, and will not collide with the wheels, which will significantly improve the stability and reliability of the system.

### 3.2. Wireless Information Transmission of the Sensors in Track Skates

After the installation of the intelligent track skates, their wireless transmission signals will be vulnerable due to the surrounding metal interferences caused by the rolling stocks. The wireless communication technology for the intelligent module of the track skates will work better for the work performance of the system. 

Different types of wireless communication technologies exhibit different characteristics. Recently, 2.4 G low-power-consumption wireless communication devices have obtained added functions of remote/proximity identification and authentication and data collection by the sensor network without the addition of any auxiliary hardware. The wireless USB recently developed by Cypress Semiconductor Corporation has compatibly combined the universal serial wireless technology, resulting in efficient and high-speed short-distance wireless transmission. With the use of the 2.4 G spectrum, the wireless USB is relatively easier to operate and consumes less power as compared to technologies such as ZigBee and Bluetooth. In particular, it is specialized in point-to-point and multipoint-to-multipoint transmission of small data packs. Since the intelligent track skate system demands the relatively small amount of data, the wireless USB technology can completely satisfy the need. We, therefore, used the wireless USB in our wireless communication module within the real-time monitoring and management system to conduct anti-runaway prevention, as shown in Figure 4.

### 3.3. Microprogrammed Control Unit Module of Intelligent Track Skates

Microprogrammed control units (MCUs) have wide applications such as intelligent instrument manufacturing, job control, electronic communications, and electronic component design, owing to their compact size, low power consumption, flexibility, and adaptability to the environment. An MCU is used as the main control chip for management and control of the intelligent track skates. An MCU needs a certain type of chip to transmit data, thus enabling the serial peripheral interface to work by combining the advantages of enough data storage space, low power consumption, strong computing power, and timing counting ability. In contrast to other chips, CYRF6936 has many advantages, including increased operating voltage range, the reduced supply current in all operating modes, higher data rate options, reduced crystal startup, synthesizer settling, and decreased link turnaround times. Therefore, to develop the intelligent track skates, we chose CYRF6936 and an ATmega168 microcontroller after comparing several MCUs regarding shape, price, and performance, to transmit data (Figure 5).

### 3.4. Battery Using Time of Intelligent Track Skates

One of the biggest difficulties in the process of developing intelligent track skates is to solve the problem of power consumption, since the performance of electronic components usually depends on the power supply. Due to the randomness of their position, a track skate normally adopts only large-capacity batteries for power supply. Hence, in such operation process for track skates, the staff need to constantly monitor the battery usage and replace batteries, which increases their workload as well as the danger to the entire system since there are additional operation steps. Thus, the batteries used for intelligent track skates must meet the safety and economy requirements. The following are the supporting calculations for the design.

a. Electricity usage of ordinary rechargeable intelligent track skates

When track skates are in a static state (not on the rail), power consumption is 40 mAh per hour. Using a 7000 mAh rechargeable lithium battery, the track states can be used for 7.1 days:

(1) $$\frac{7000\;\mathrm{mAh}}{40\;\mathrm{mAh}\;*\;24\;h}=7.1\;d$$

When intelligent track skates are in the state of their largest power consumption (in check wheel), power consumption is 180 mAh per hour. Using a 7000 mAh rechargeable lithium battery, the skates can be used for 1.6 days:

(2) $$\frac{7000\;\mathrm{mAh}}{180\;\mathrm{mAh}\;*\;24\;h}=1.6\;d$$

Thus, the service time of the battery of intelligent track skates is just 1.6–7.1 days on average.

b. Electricity using the intelligent track skates under the control of MCUs

When a track skate is in a static state (not on the track) under MCU control, it works once in 1 s and the power consumption is 0.08 mAh per hour. Using a 7000 mAh lithium battery, the track skate can be used for 9.9 years:

(3) $$\frac{7000\;\mathrm{mAh}}{0.08\;\mathrm{mAh}\;*\;24\;h\;*\;365\;d}=9.9\;\mathrm{year}$$

The intelligent control of the circuit within an intelligent track skate works every 10 s as a sleep-and-work cycle. When the intelligent track skate is in the state of its largest power consumption (in check wheel), within each 10-s operation cycle, the intelligent track skate will work for 20 ms every time. Within this 20 ms, 5 ms are used for launching the signal and 15 ms are used for monitoring the signal. The current would be 200 mA for the emission state and 40 mA for the listening state. Thus, the accumulative transmitting time is 40 ms per minute (launching 20 times) and the accumulative monitoring signal time is 200 ms.

In this case, the electricity consumption is 0.0044 mAh per min:

(4) $$\frac{40}{1000\;*\;3600}\;h\;*\;200\;\mathrm{mA}\;+\;\frac{200}{1000\;*\;3600}\;h\;*\;40\;\mathrm{mA}=0.0044\;\mathrm{mAh}$$

The electricity consumption is 2336 mAh per year:

(5) 0.0044 mAh ∗ 60min ∗ 24h ∗ 365 d = 2336 mAh

Therefore, the usage time for batteries whose capacity is 7000 mAh can reach nearly three years:

(6) $$\frac{7000}{2336}=2.99\;\mathrm{year}$$

In the railway industry, the lifetime of traditional track skates does not exceed three years because of abrasion. Hence, this design of low-power intelligent track skates ensures increased operation time without changing the battery during the lifetime of the track skates.

## 4. Design of Real-Time Monitoring and Management System

### 4.1. Signal Reader

**Figure 8:**
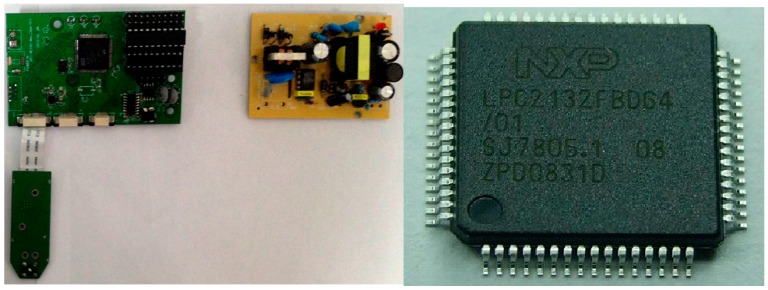
Signal Reader.

A signal reader is responsible for sending information received from the sensor to the database, and comprises a power supply module, the main control module, and a wireless transceiver module. Signal devices can be placed in a fixed position concerning the external power supply. LPC2131 microcontrollers are based on a 32/16-bit ARM7TDMI-S CPU with real-time emulation and embedded trace support, which combines the microcontroller with high-speed Flash memory. In addition, the compact size and low power consumption of LPC2131 make it ideal for applications where miniaturization is a key requirement, such as access control and point-of-sale. Therefore, we selected the LPC2132 microcontroller as the signal reader master control chip, see Figure 8.

### 4.2. Design of Database Station

The base station is responsible for receiving a signal sent by the wireless information and transmitting it to the monitoring system. Similar to the signal reader, the base station also comprises three parts: the power supply module, main control module, and wireless transceiver module. We used CYRF6936 and LPC2132 microcontroller chips as the wireless communication module and the main control chip, respectively.

**Figure 9:**
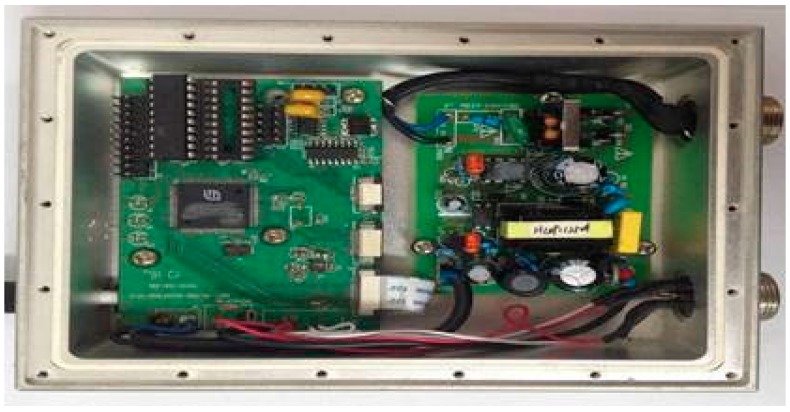
Internal structure of data station.

The database station transmits information from the monitoring system through cables based on the RS485 communication protocol, which has the electrical characteristics of drivers and receivers for use inserial communicationsystems. The RS485 communication protocol was released after the RS422 communication protocol, and uses a half-duplex communication mode. During the actual operation, a driver can drive at least 32 receivers and a larger number when using a receiver with higher impedance. Now, since the full-duplex RS485 drivers/receivers can be applied to the RS422 network, most full-duplex 485 drivers/receivers are identified by RS422/485. In addition, RS485 has the stronger anti-interference ability, longer communication distance, and higher communication rate compared to RS232 (see Figure 9).

### 4.3. Human Machine Interface

The human–machine interface, developed using C# language, which perfectly cooperates with DirectX imaging, refreshes the efficiency of system interface revolutionarily and is more exquisite and user-friendly. Compared to traditional GDI and OpenGL imaging, DirectX significantly reduces the burden of the CPU based on the uses of the GPU, resulting in higher efficiency, speed, and stability of the monitoring system and thread code.

**Figure 10:**
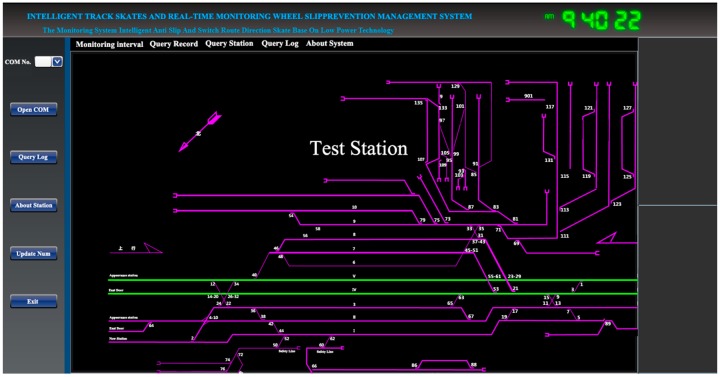
Monitoring interface.

A monitoring system using active alerts and asynchronous loop detection mechanism realizes an efficient serial data transmission. Using the method of timely memory clearance and providing buffer data flow, the monitoring system prevents the data updating. Compared to other existing open-source analysis methods, this method is more efficient and stable. It also decreases the computer blue screen phenomenon when a computer interacts with the hardware (see Figure 10).

An intelligent monitoring system forms a complete set of the track skate system, assists the station staff in managing the track skates, and guarantees the safety and efficiency of the operating conditions. The monitoring system realizes the following functions:

- It uses a simple and clear user interface for better human–computer interaction, making it convenient for operators and shunting teams to operate. It also improves the flexibility for information searching and the security and reliability of data storage.
- It can help operators finishing initialization operations in yard status setting, track status setting, information display settings, and serial port settings. It is convenient for operators to set the yard status, to switch the yard to a serial port, to look at the notes, and to update the technical components.
- The system can monitor the real-time status of intelligent track skates and trains, displaying the real-time status of the track skates and the number and types of rolling stocks at the station, providing historical data searching and log querying, which makes it easier for operators to use the data.

## 5. Practical Value of the Intelligent Track Skates

With the application of advanced and reliable modern communication and information management, ARPS has changed the traditional and outdated process of Chinese railway anti-runaway prevention, which used to lack technical measures for safety management. 

This system saves labor cost significantly and avoids accidents caused by human errors in shunting operations. Since the highest power of a base station is only 5 W, the intelligent track skates can work continuously for more than three years without charging when installing at an appropriate location on the platform. The effective communication distance between the track skates and the database terminal covers a circular area of 750 m radius, and can be applied to most railway stations in China with significant economic and social benefits. This system was successfully tested and applied in 2014 at Sunjia station in Harbin Railway Bureau. We first tested the devices for 20 days in February, using 329 records. The conditions and the contents tested are summarized in Table 1. We mainly tested two aspects of the effectiveness of the intelligent track skates. First, the reliability of the communication distance. We tried the maximum distance, if the database station could receive data from the track skates continuously and steadily, which would confirm that the tested distance should be in the effective distance of the wireless communication range. We used distances of 1000, 900, 800, 750, and 700 m, and found that the system can work well only when the communication distance is within 750 m. Second, we placed and moved the track skates continuously to test whether the database terminal could receive the data and indicate the status of the track skates timely and correctly, and we found that the system was effective as all operations on the track skates were reflected on the screen in the database station within 3 s. Then, 5613 practical records obtained from 225 days were used. The results shown in Table 2 confirm that the system showed 100% accuracy in reflecting the status of the track skates. In addition, the communication distances and reliabilities of the system met the practical demands.

## 6. Conclusions

ARPS is the most widely used anti-slipping apparatus at railway stations. Using the existing track skates, the operators are unable to obtain real-time dynamic information about the usage status of track skates. In the case the track skates are stolen, the on-site staff violate the rules and regulations, or a track skate is not placed at the exact designated position, accidents are likely to occur. Confronted with problems in the operation process of track skates, we developed an intelligent track skate equipped with sensors and real-time monitoring and management system. The proposed system can send real-time on-track information of the track skates status, coping with the problems of the current operation process of track skates, which mainly relies on human intervention and is prone to accidents caused by improper installation or abnormal situations. The proposed system can reduce the loss or improper use/installation of track skates, preventing the anti-runaway of railway rolling stocks, to enhance safety and avoid errors due to negligence in operation. 

Harbin Railway Bureau has 123 heavy-workload railway stations within 406 stations in total, which requires continuous real-time monitoring for ensuring the safety of anti-runaway operation for parked rolling stocks. The system can be used for 18 railway bureaus, specifically for about 2000 stations across China, to improve the safety management of railway stations.

The real-time monitoring and management system of ARPS still has some issues that need to be improved. First, this system is still unable to help track skates obtain accurate positions, even though the precise locations of track skates on the tracks are not absolutely necessary under the existing operation process. If intelligent track skates can send more effective information, it will undoubtedly make the whole system safer for the railway. Second, the proposed intelligent track skates can only adapt to a communication distance of within 750m in China, and cannot be applied in North America as many classification yard tracks there are over 1000 m in length. Thus, we need to try other sensors with a longer communicate distance to realize the balance between cost and efficiency. Finally, the function of recording and analyzing the status of anti-runaway prevention should be carried out automatically in the future.

## Figures and Tables

**Table 1:** Conditions and contents tested.

Attributes	Contents
Location	Sunjia station
Circumstances	Real-world transport circumstances
Tested contents	Reliable communicating distances, Idle current, emission current, Receive current
Devices	Computers (terminal), Constant—Current DC power supply, Oscillometer, Multimeter, Intelligent track skates (two), Signal reader (two), Database station (one)
Test results	All the result meet the design requirements

**Table 2:** Results of the Test and Practices.

Electronic Tag	Standby Current (µA)	Receive Current (mA)	Emission Current (mA)	Maximum Communicate Distance (m)
10111032000001	11	38	215	750
10111032000002	8	40	206	750
